# Three-dimensional and real-scale modeling of flow regimes in dense snow avalanches

**DOI:** 10.1007/s10346-021-01692-8

**Published:** 2021-07-29

**Authors:** Xingyue Li, Betty Sovilla, Chenfanfu Jiang, Johan Gaume

**Affiliations:** 1 School of Architecture, Civil and Environmental Engineering, Swiss Federal Institute of Technology, Lausanne, Switzerland; 2 WSL Institute for Snow and Avalanche Research SLF, Davos, Switzerland; 3 Computer and Information Science Department, University of Pennsylvania, Philadelphia, USA

**Keywords:** Snow avalanche, 3D real-scale modeling, Material point method, Flow regime

## Abstract

Snow avalanches cause fatalities and economic loss worldwide and are one of the most dangerous gravitational hazards in mountainous regions. Various flow behaviors have been reported in snow avalanches, making them challenging to be thoroughly understood and mitigated. Existing popular numerical approaches for modeling snow avalanches predominantly adopt depth-averaged models, which are computationally efficient but fail to capture important features along the flow depth direction such as densification and granulation. This study applies a three-dimensional (3D) material point method (MPM) to explore snow avalanches in different regimes on a complex real terrain. Flow features of the snow avalanches from release to deposition are comprehensively characterized for identification of the different regimes. In particular, brittle and ductile fractures are identified in the different modeled avalanches shortly after their release. During the flow, the analysis of local snow density variation reveals that snow granulation requires an appropriate combination of snow fracture and compaction. In contrast, cohesionless granular flows and plug flows are mainly governed by expansion and compaction hardening, respectively. Distinct textures of avalanche deposits are characterized, including a smooth surface, rough surfaces with snow granules, as well as a surface showing compacting shear planes often reported in wet snow avalanche deposits. Finally, the MPM modeling is verified with a real snow avalanche that occurred at Vallée de la Sionne, Switzerland. The MPM framework has been proven as a promising numerical tool for exploring complex behavior of a wide range of snow avalanches in different regimes to better understand avalanche dynamics. In the future, this framework can be extended to study other types of gravitational mass movements such as rock/glacier avalanches and debris flows with implementation of modified constitutive laws.

**Supplementary Information:**

The online version contains supplementary material available at 10.1007/s10346-021-01692-8.

## Introduction

Gravitational mass movements cause tremendous damages in mountainous regions all over the world. Although they are all driven by gravity, their behavior may differ much from one another due to notably different constitutions (e.g., rock, soil, water, ice) and mechanisms of motion (e.g., fall, slide, flow). Taking landslides as an example, 32 types have been classified according to material and movement types (Hungr et al. 2014), including rock/ice avalanche, debris flow, and soil creep. Like other types of gravitational mass movements, snow avalanches can behave differently throughout their movements from triggering to deposition, under the effect of snow properties, weather conditions (e.g., air temperature and wind velocity), terrain features, etc. At the release of snow avalanches, there are two major patterns observed in the field: a fracture line and a point release which distinguish slab avalanches and loose snow avalanches, respectively. These distinct release types are primarily controlled by snow cohesion. Relatively cohesionless snow is prone to produce a point release, while more cohesive snow tends to generate a fracture line (Schweizer et al. 2003). During the flowing process, three layers can be characterized based on flow density, contacts between snow particles, and interactions between snow particles and air (Ancey 2001; Gauer et al. 2008; Köhler et al. 2018b). If snow density is high and contacts between snow particles are numerous, a dense layer is formed. With the reduction of particle-particle contacts, a fluidized layer appears on top of the dense layer. Furthermore, if the flow density is low and the particle-air interactions dominate, a suspension layer occurs. In addition to the different release and flowing characteristics, distinct textures of snow avalanche deposits have been observed as well. Based on surface roughness of avalanche deposits, coarse deposits (including angular blocks and rounded clods) and fine deposits were classified (ICSI-IAHS 1981). Recently, (Issler et al. 2020) identified avalanche deposits including blocky and sharply bounded deposit, snow clods, and fine-grained snow. These diverse behaviors make snow avalanches fantastic phenomena which involve challenging research questions with substantial societal and economical impacts.

Based on field observations and measurements, snow avalanches with different characteristics have been classified into various flow regimes. A recent study from (Köhler et al. 2018b) identified seven flow regimes, by differentiating between starting, flowing, and stopping signatures. Specifically, for dense snow avalanches, four flow regimes were distinguished, namely cold dense, warm shear, sliding slab, and warm plug. Avalanches in the cold dense and warm shear regimes behave like fluids or cohesionless granular flows, which are notably sheared throughout the flow depth direction. The snow granules in a warm shear flow are larger than the snow particles in a cold dense flow, as granulation occurs for temperature close to 0°C. Both the sliding slab and warm plug regimes resemble solid-like objects sliding down the slope. A sliding slab avalanche can be distinguished by its brittle slab fractures, while a warm plug avalanche demonstrates ductile behavior.

While field investigations offer valuable and realistic data of macro flow behaviors of snow avalanches, numerical analysis can provide useful insights in both temporal and spatial scales and help reveal underpinning physics. Current popular numerical approaches for modeling snow avalanches predominantly adopt depth-averaged models (Christen et al. 2010; Medina et al. 2008; Mergili et al. 2017; Naaim et al. 2013; Rauter et al. 2018). Based on shallow-water theory, depth-averaged methods neglect the spatial variation in flow depth direction to facilitate efficient computation. Nevertheless, it has been recognized that important features along the flow depth, such as snow granulation and compaction/densification, can affect avalanche velocity, runout, and impact force on obstacles (Eglit et al. 2020; Gaume et al. 2019). In particular, snow density variations are rarely accounted for in numerical and theoretical analyses of snow avalanches but directly affect the impact force on obstacles and are crucial in mitigating avalanche impacts. Only limited studies considered the streamwise evolution of snow density in avalanches (Buser and Bartelt 2011; Buser and Bartelt 2015).

Both discrete and continuous numerical methods have been developed to study avalanches. The discrete element method (DEM) assumes a discontinuous granular medium, which facilitates the recovery of microscale features and offers insights into local rheology of an avalanche (Kneib et al. 2019; Kyburz et al. 2020; Macaulay and Rognon 2020). However, the high computational cost of DEM limits its application to real-scale investigations. Alternatively, continuum approaches are suitable for modeling large-scale systems and capturing their flow features in an efficient way. Mesh-free continuum approaches, such as particle finite element method (PFEM), smooth particle hydrodynamics (SPH), and material point method (MPM), have become increasingly popular in exploring granular avalanches, as they can readily handle large-deformation problems without expensive remeshing (Abdelrazek et al. 2014; Gaume et al. 2018; Salazar et al. 2016). The character of these approaches facilitates solving processes involving collisions, fractures, and large deformations.

Different flow regimes of dense snow avalanches have been previously explored with two-dimensional (2D) MPM (Li et al. 2020), where sensitivity analyses of snow property and slope geometry (i.e., slope angle, path length) were conducted. This earlier study offers insights into the mechanical origin of the dynamic behavior of snow avalanches and their flow regime transitions. Nonetheless, ideal slopes were adopted with neglection of terrain irregularity. Meanwhile, to facilitate efficient computation, variations in flow width direction were not considered.

In this study, our goal is to simulate and analyze, from release to deposition, the mechanics of distinct flow regimes reported in real-scale snow avalanche experiments. To go beyond the limitations of existing 2D approaches, we propose a three-dimensional (3D) MPM and an elastoplastic constitutive law for porous cohesive materials. To recover the natural boundary conditions of the avalanches, the real terrain of Vallée de la Sionne, a Swiss test site for avalanche experiments (Ammann 1999), is implemented. By virtue of the elastoplastic model that retrieves the mixed-mode failure of snow, the failure patterns after avalanche release are explored for the modeled snow avalanches. Furthermore, avalanche density variation, which plays a crucial role in altering flow regime, run-out distance, and impact pressure (Buser and Bartelt 2015; Kyburz et al. 2020; Issler and Gauer 2008), are scrutinized throughout the flowing process. In addition, snow avalanche deposits are analyzed, to provide meaningful inferences on flow mechanisms and flow regimes (Issler et al. 2020; Issler et al. 2008). Finally, we compare a real snow avalanche with a simulation case for verification of our modeling framework.

## Methodology

### The material point method

**Fig. 1:**
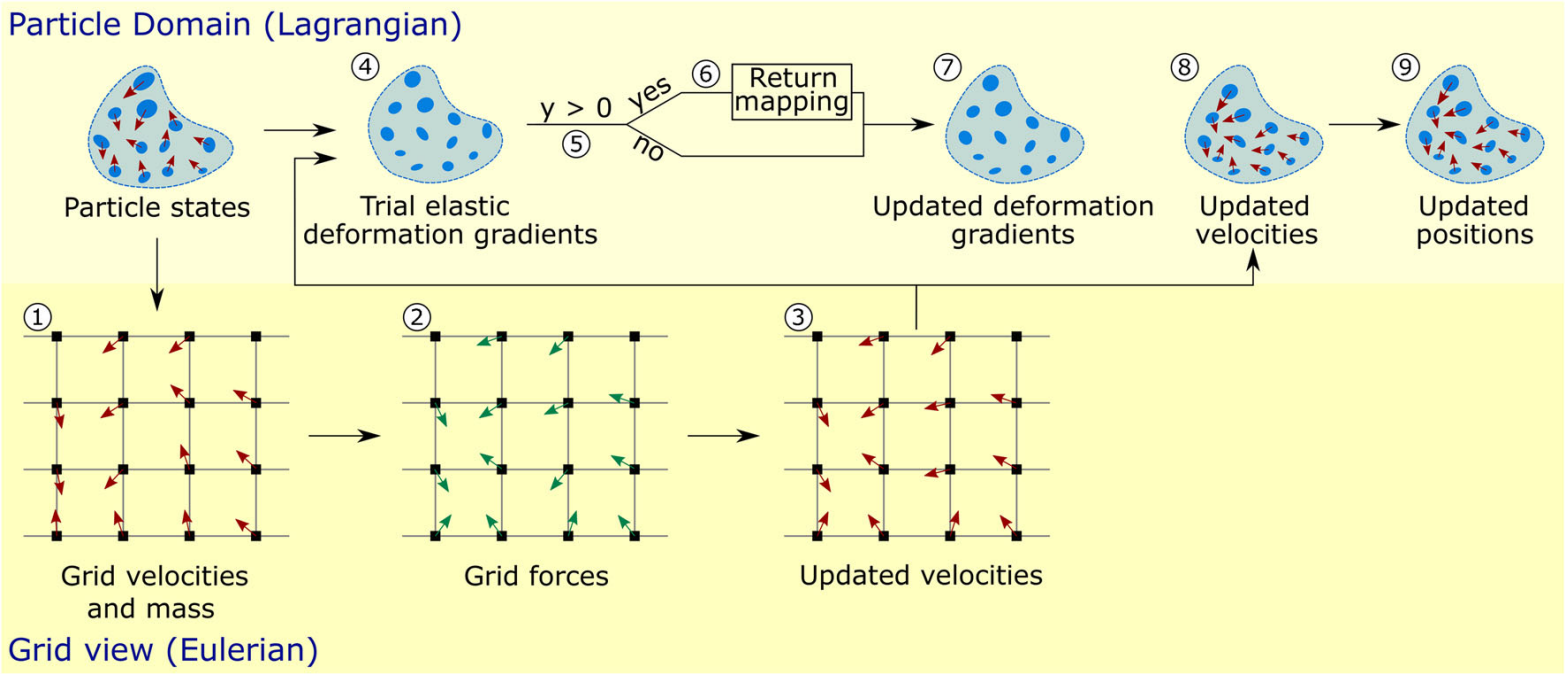
Overview of the MPM algorithm. Modified based on (Gaume et al. 2018; Stomakhin et al. 2013)

To efficiently and effectively model snow avalanches involving large deformations, fractures, and collisions, the material point method (MPM) is used in this study. MPM assumes a material as a continuum and discretizes it into Lagrangian particles (material points). The states of the particles, including mass, position, momentum, and deformation gradient, are updated by adopting Eulerian grids, on which the motion of the particles is solved. Following (Gaume et al. 2018; Stomakhin et al. 2013), this study adopts the explicit MPM algorithm as illustrated in Fig. 1. In the first step, the mass and velocity of particles are transferred to the grid to get grid mass and velocity. Then, the force at each grid node is computed (Step 2) considering the elastic stresses of the particles close to the node, during which a constitutive model of snow (detailed in the “Constitutive snow model” subsection) is applied. The grid velocity can be further updated (Step 3) under the constraint of momentum conservation (Eq. 2). Assuming that particles deform in a purely elastic way, their trial elastic deformation gradients are computed (Step 4), and the yield condition in Eq. 4 is checked (Step 5). If the assumption of purely elastic deformation is false (y > 0), return mapping is performed (Step 6) before deformation gradients are updated (Step 7). Otherwise (y ≤ 0), the trial elastic deformation gradients (at Step 4) are directly updated as the new ones (at Step 7). Finally, the updated grid velocities are transferred back to the particles to obtain the updated particle velocities (Step 8) and positions (Step 9).

Particularly, the particle motion satisfies the following mass and momentum conservation:

1 $$ \frac{D\rho}{D t}+\rho \nabla \cdotp \boldsymbol{v}=0 $$

2 $$ \rho \frac{D\boldsymbol{v}}{Dt}=\nabla \cdotp \boldsymbol{\sigma} +\rho \boldsymbol{g} $$

where *ρ* denotes density, *t* is time, ***v*** is velocity, ***σ*** is the Cauchy stress, and ***g*** is the gravitational acceleration. In MPM, the mass of each Lagrangian particles does not change, which naturally guarantees the balance of mass. The momentum conservation in Eq. (2) is solved with the discretization of its weak form on a regular background Eulerian mesh. Following (Gaume et al. 2018), we employ the explicit MPM algorithm and a symplectic Euler time integrator (Gaume et al. 2018; Stomakhin et al. 2013; Jiang et al. 2016). It is worth noting that, compared to (Gaume et al. 2018), angular momentum of the particles is preserved during the grid-to-particle transfer in this study by using the Affine Particle-In-Cell (APIC) method (Jiang et al. 2015).

### Constitutive snow model

A large-strain elastoplastic constitutive model is applied for the modeling of snow (Gaume et al. 2018), which relates the Cauchy stress ***σ*** in Eq. 2 to the strain as follows:

3 $$ \boldsymbol{\sigma} =\frac{1}{J}\frac{\partial \varPsi }{\partial {\boldsymbol{F}}_E}{\boldsymbol{F}}_E^T $$

where *Ψ* is the elastoplastic potential energy density, ***F***_*E*_ represents the elastic part of the deformation gradient ***F***, and *J* = det(***F***). The elastoplastic model is composed of a mixed-mode shear-compression yield surface, a hardening law, and an associative flow rule. The main characteristics of the three key components are revisited as follows.

**Fig. 2:**
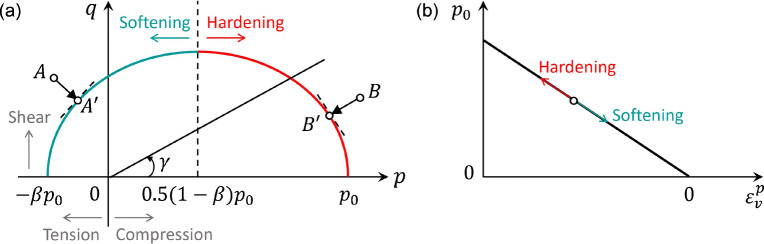
**a** Adopted yield surface in the *p-q* space. **b** Illustration of the hardening law in Eq. 5

As illustrated in Fig. 2a, the adopted yield surface is a cohesive Cam-clay yield criterion, which recovers the mixed-mode nature of snow failure (including tensile, shear, and compression failure modes) revealed from experiments (Reiweger et al. 2015) and simulations (Chandel et al. 2015; Hagenmuller et al. 2015; Srivastava et al. 2017), and is defined as:

4 $$ y\left(p,q\right)=\left(1+2\beta \right){q}^2+{M}^2\left(p+\beta {p}_0\right)\left(p-{p}_0\right) $$

*p* =  − tr(***τ***)/*d* is the pressure calculated from the Kirchhoff stress tensor ***τ***, and the dimension *d*. *p* > 0 and *p* < 0, respectively, indicate compression and tension. The Mises stress *q* is defined as *q* = (3/2 ***s*** : ***s***)^1/2^, where ***s*** is the deviatoric stress tensor equaling to ***τ*** + *p****I***, and ***I*** is the identity matrix. *q* > 0 hints shear. The pre-consolidation pressure is denoted as *p*_0_, and *βp*_0_ denotes the isotropic tensile strength, where *β* controls the amount of cohesion. The internal friction is reflected by *M*, which is the slope of the critical state line. In Fig. 2a, *γ* represents the loading angle, and thus *γ* = 0°, 90°, and 180° indicate pure compression, pure shear, and pure tension, respectively. When 0^∘^ < *γ* < 90^∘^, a snow particle sustains both compression and shear. If 90^∘^ < *γ* < 180^∘^, a snow particle is under tension and shear.

Based on the current *p-q* state of the material and Hooke’s law (St Venant-Kirchhoff Hencky strain), a trial *p-q* state can be obtained with the assumption of purely elastic deformation. The yield condition in Eq. 4 can then be checked. If the trial *p-q* state is inside or on the yield surface (i.e., *y*(*p*, *q*) ≤ 0), it is updated as the new *p-q* state. Otherwise (*y*(*p*, *q*) > 0), the trial *p-q* state is non-admissible (e.g., Points *A* and *B* in Fig. 2a), and plastic behavior needs to be involved, which requires a flow rule to project the trial *p-q* state (e.g., Points *A* and *B*) back to the yield surface (e.g., Points *A*^′^ and *B*^′^) and a hardening law to adjust the yield surface.

In case of the occurrence of plastic deformation, the following hardening law (illustrated in Fig. 2b) is employed to account for the hardening or softening of the material by expanding or shrinking the yield surface:

5 $$ {p}_0=K\sinh \left(\xi \max \left(-{\epsilon}_v^p,0\right)\right) $$

where *K* is the bulk modulus, *ξ* is the hardening factor, and ${\epsilon}_v^p$ is the volumetric plastic strain. It is assumed that the hardening and softening solely depend on ${\epsilon}_v^p$. When the non-admissible *p-q* state is on the left side of the apex of the yield surface (e.g., Point *A*), the plastic volume increases (${\dot{\epsilon}}_v^p>0$), and *p*_0_ decreases, resulting in softening and allowing fracture under tension. Otherwise (e.g., Point *B*), the plastic deformation is compressive (${\dot{\epsilon}}_v^p<0$), and *p*_0_ increases, leading to hardening and more elastic responses resisting compression.

Referring to (Gaume et al. 2018), we use an associative plastic flow rule (Simo and Meschke 1993; Simo 1992). As demonstrated in Fig. 2a, the projection path from Point *A* (or *B*) to Point *A*^′^ (or *B*^′^) is perpendicular to the tangent line to the yield surface at Point *A*^′^ (or *B*^′^). By following the principle of maximum plastic dissipation, the flow rule gives a plastic flow that maximizes the plastic dissipation rate. Combined with the adopted flow rule, our plastic model fully satisfies the second law of thermodynamics. The elliptic shape of our yield surface and the associative flow rule have been recently shown to be appropriate for the simulation of porous brittle solids like snow (Ritter et al. 2020). More details of the flow rule can be found in (Gaume et al. 2018).

### Model setup

**Fig. 3:**
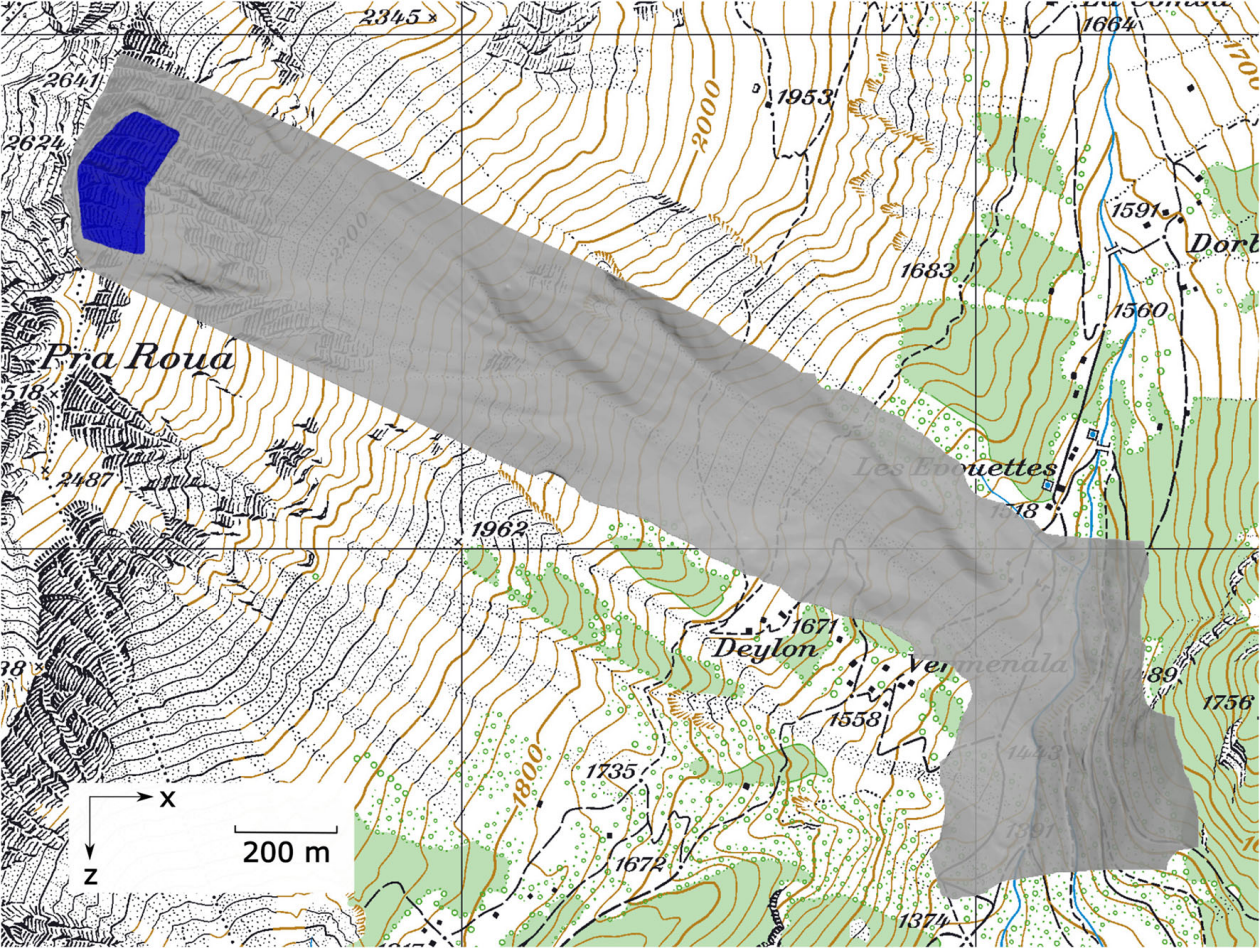
Top view of the MPM simulation setup

This study adopts the terrain of the Vallée de la Sionne test site in Switzerland, where field data have been collected for the investigation of snow avalanche dynamics over the last 20 years. These data offer useful information to understand snow avalanches and are also key for model validation and calibration (Köhler et al. 2018b; Ammann 1999; Sovilla et al. 2006). Figure 3 shows the top view of the MPM simulation set up. At the top of the simulated terrain in gray, a snow volume in blue is initially placed at the release zone and will flow down under gravity. To save computational cost, only the snow in the release zone is simulated, while the snow cover on the terrain is not accounted for. The slope angle and curvature maps of the modeled terrain are attached in the supplement. The location and size of the release zone are adapted from a real snow avalanche artificially triggered on 7 February 2003 (Sovilla et al. 2006), which serves as a benchmark case and will be quantitatively compared with our MPM simulation. The snow depth in the release zone is 1.05 m in the MPM modeling, referring to the average fracture height of the real snow avalanche. The resolution of the digital terrain model adopted in this study is 0.5 m.

The background mesh in the simulation is composed of cubes with identical size (0.5 m × 0.5 m × 0.5 m). Each grid element is filled with 8 material points, and the total number of material points in each simulation is about 1.9 million. The time step is 2 × 10^−3^ s, determined with constraints based on the CFL condition and the elastic wave speed. The computational time for one simulation with real time of 100 s is around 5 h on a 36-core Intel i9 CPU (3.0 GHz) desktop computer.

It should be noted that the grid size and orientation are fixed for the simulations in this study. We show in the supplement that the simulation results are not affected by the grid size and orientation. Note that no complex regularization scheme (e.g., Cosserat continuum or second order gradient (Desrues et al. 2019)) has been implemented because the physical length scale related to fracture is of the order of the grain size (< 1 cm), which is significantly lower than any reasonable resolution for a large-scale simulation. Yet, to prevent mesh dependency, one can adjust the hardening factor in order to dissipate the same amount of energy during fracture for different mesh resolutions (Gaume et al. 2018; Mahajan et al. 2010; Sulsky and Peterson 2011).

As listed in Table 1, five cases with different snow and slope properties are investigated in this study. Cases I∼IV share the same slope property and are designed to examine the behavior of avalanches with different snow properties, while case V serves as a verification case to be compared with the real avalanche happened on 7 February 2003. The four sets of snow parameters in cases I∼IV are adapted from 2D MPM simulations with various combinations of model parameters reflecting generic snow properties, namely the friction coefficient *M*, the tension/compression ratio *β*, the hardening factor *ξ*, and the initial consolidation pressure ${p}_0^{ini}$ (Li et al. 2020). Note that due to the scarcity of snow triaxial tests (Desrues et al. 1980; Scapozza and Bartelt 2003) required to calibrate a model based on critical state soil mechanics, we use generic model parameters that were proven appropriate to reproduce reported avalanche flow regimes. With the specific setup of the 2D simulations in (Li et al. 2020), the snow properties in cases I∼IV gave four typical flow regimes of dense snow avalanches, which are cold dense, warm shear, sliding slab, and warm plug regimes from case I to case IV. Although the four regimes were clearly characterized in the 2D simulations using the four sets of snow parameters, the applied slope was assumed to be ideal without consideration of terrain irregularity. Given the current 3D complex terrain in Fig. 3, the flow regime may differ even with the same sets of snow parameters. The snow and slope properties in case V are determined based on back calculation of the real snow avalanche. In the following section, we firstly explore distinct features of the snow avalanches in cases I∼IV from their release to deposition and then discuss the verification case V.

**Table 1:** Parameters adopted in the MPM simulations

		Case I	Case II	Case III	Case IV	Case V
Snow property	Friction coefficient *M*	0.5	1.5	1.5	0.5	0.7
	Tension/compression ratio *β*	0	0.3	0.5	1.0	0.2
	Hardening factor *ξ*	1	1	1	0.1	0.002
	Initial consolidation pressure ${p}_0^{ini}$ (kPa)	3	30	42	12	3
	Initial density *ρ*_0_ (kg/m^3^)	250	250	250	250	200
	Young’s modulus (MPa)	3	3	3	3	3
	Poisson’s ratio	0.3	0.3	0.3	0.3	0.3
Slope property	Friction coefficient *μ*	0.47	0.47	0.47	0.47	0.49

## Results

### Snow avalanches in different flow regimes

As detailed in the “Introduction” section, flow regimes of snow avalanches have been identified based on their release, flowing, and deposition behavior (Schweizer et al. 2003; Ancey 2001; Gauer et al. 2008; Köhler et al. 2018b; Issler et al. 2020). Aiming at simulating the variety of flow regimes reported in dense snow avalanches and offering quantitative proof for the regime characterization, we analyze the features of the snow avalanches in cases I∼IV from release to deposition, including failure pattern after avalanche release, density variation during the flow, and deposition texture. In particular, the failure pattern is associated with the stress state of snow particles, which helps distinguish the amount of fractures developed in snow avalanches and the type of the fractures. Moreover, both temporal and spatial variations of density are investigated, shedding light on the expansion and compaction of snow.

#### Failure pattern after avalanche release

**Fig. 4:**
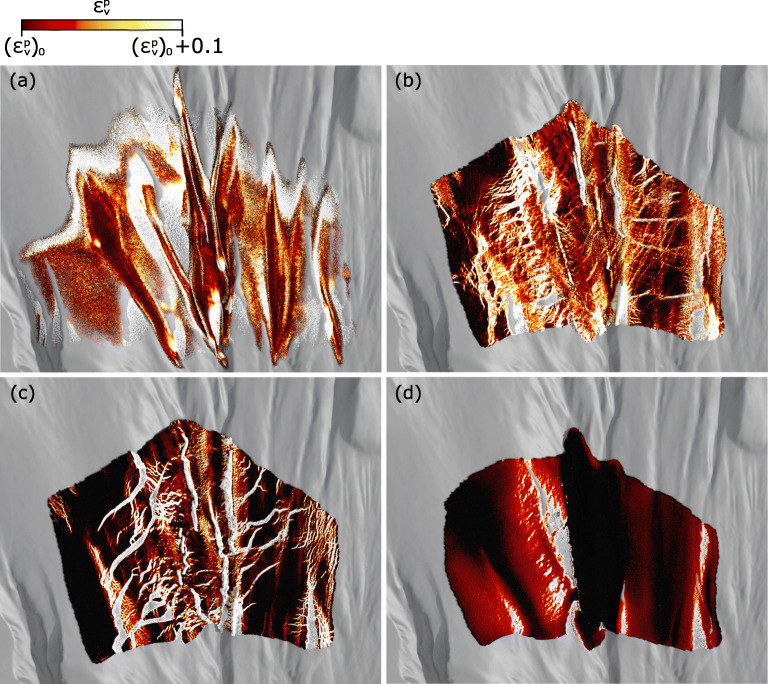
Volumetric plastic strain of the four flows at 10 s on the real terrain in gray. **a** case I: ${\left({\boldsymbol{\epsilon}}_{\boldsymbol{v}}^{\boldsymbol{p}}\right)}_{\mathbf{0}}$ = −0.0012; **b** case II: ${\left({\boldsymbol{\epsilon}}_{\boldsymbol{v}}^{\boldsymbol{p}}\right)}_{\mathbf{0}}$ = −0.0120; **c** case III: ${\left({\boldsymbol{\epsilon}}_{\boldsymbol{v}}^{\boldsymbol{p}}\right)}_{\mathbf{0}}$ = −0.0168; **d** case IV: ${\left({\boldsymbol{\epsilon}}_{\boldsymbol{v}}^{\boldsymbol{p}}\right)}_{\mathbf{0}}$ = −0.0480

To explore different failure patterns after the release of the snow avalanches, the volumetric plastic strain of the snow is shown in Fig. 4. The initial volumetric plastic strain ${\left({\epsilon}_v^p\right)}_0$ is used to determine the initial consolidation pressure of the snow (using Eq. 5). The illustrated range of ${\epsilon}_v^p$ in Fig. 4 is selected to highlight the developed fractures. The avalanche in case I (Fig. 4a) behaves as a cohesionless granular flow, where multiple branches are formed along the concave parts of the terrain. No clear pattern of fractures is observed as the snow has no cohesion (*β* = 0) and thus no tensile strength (*βp*_0_ = 0). The avalanches in cases II and III (Fig. 4b and c) share similar characteristics, both of which largely keep the initial shape of the release zone. In addition, sharp fractures appear in cases II and III, showing the typical signature of a slab avalanche where a snow slab breaks into pieces after release. Compared to case III, the fractures in case II are denser and thinner, and the growth of plastic volume is more extensively distributed (reflected by the lighter color in Fig. 4b). This is tightly related to the smaller cohesion and tensile strength of the snow in case II (*β* = 0.3; *βp*_0_ = 9 kPa) in comparison to that in case III (*β* = 0.5; *βp*_0_ = 21 kPa). Analogous to cases II and III, the avalanche in case IV (Fig. 4d) has fractures developed as well. Nevertheless, the fractures in case IV are ductile in contrast to the brittle fractures in cases II and III. Note that the brittleness of the snow is mainly controlled by the hardening factor *ξ*. As the snow in case IV has a smaller *ξ* (see Table 1), it deforms in a ductile manner. This ductile signature of the avalanche in case IV is akin to a plug flow.

**Fig. 5:**
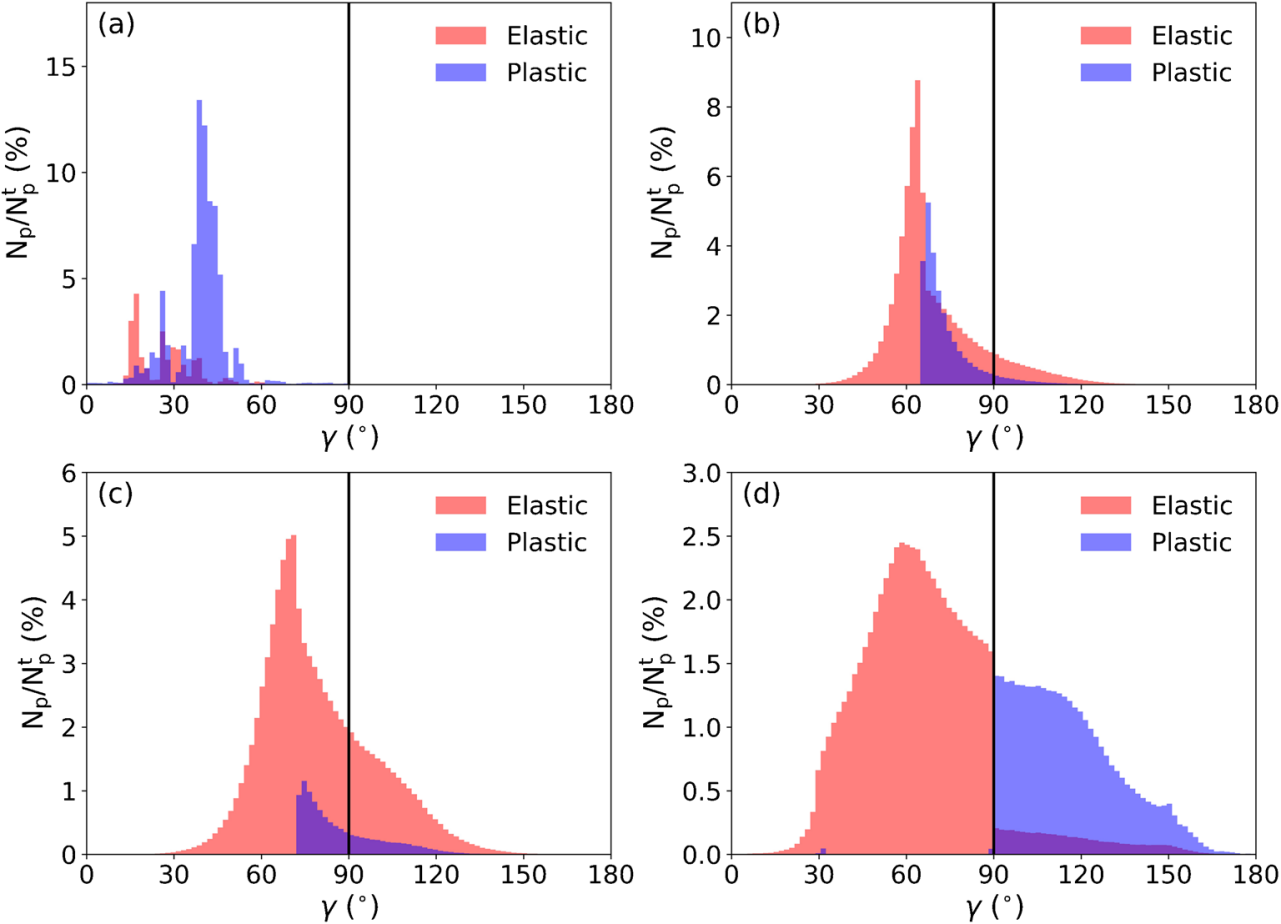
Distribution of particle stress state at 10 s. **N**_**p**_ and ${N}_p^t$ are the particle number at each interval of ***γ*** and the total particle number, respectively. The interval of ***γ*** is 1.8**°**. **a** case I; **b** case II; **c** case III; **d** case IV. Red and blue denote distributions of elastic and plastic particles, respectively. Purple shows the overlapped part of the distributions of elastic and plastic particles

To offer quantitative support on the analyses of the observed fractures, the distribution of particle stress state is illustrated in Fig. 5. The entire domain of *γ* (i.e., [0°, 180°] as shown in Fig. 2a) is divided into 100 intervals with an interval width of 1.8°. *N*_*p*_ and ${N}_p^t$ are the particle number at each interval of *γ* and the total number of particles with a non-zero pressure *p*, respectively.

The ratio of failed particles in snow can reflect the intensity of developed fractures, which is indicated by the area of the blue zone compared to that of the red zone in Fig. 5. According to the simulation data, the ratio of the plastic particles from case I to case IV is 77.2%, 25.8%, 10.4%, and 34.0%. The highest ratio in case I is consistent with the lowest strength of the snow, leading to the failed snow particles distributed extensively within the flow and consequently the absence of fracture pattern in Fig. 4a. The ratio of plastic particles in case II is around twice of that in case III, consistent with the denser fracture network observed in case II. From visual inspection of Fig. 4, less fractures are noted in case IV compared to cases II and III. However, more plastic particles occur in case IV. This is due to the different types of the fractures. The brittle fractures in cases II and III happen without apparent deformation before the breakages. In contrast, the ductile fractures in case IV induce notable deformation before they appear, resulting in the more plastic particles in case IV. Indeed, the stress state of the plastic particles in cases II and III differs much from that in case IV. As shown in Fig. 5b and c, there are both plastic particles under compression and shear (*γ* < 90^∘^) and under tension and shear (*γ* > 90^∘^) in cases II and III. In comparison, the plastic particles in case IV are primarily under tension and shear (*γ* > 90^∘^), reflecting numerous particles failed in tension and shear for the formation of the ductile fractures. Note all the particles in case I sustain compression and shear without the occurrence of tension (*γ* < 90^∘^ in Fig. 5a), since the snow has no tensile strength.

#### Density variation during the flowing process

**Fig. 6:**
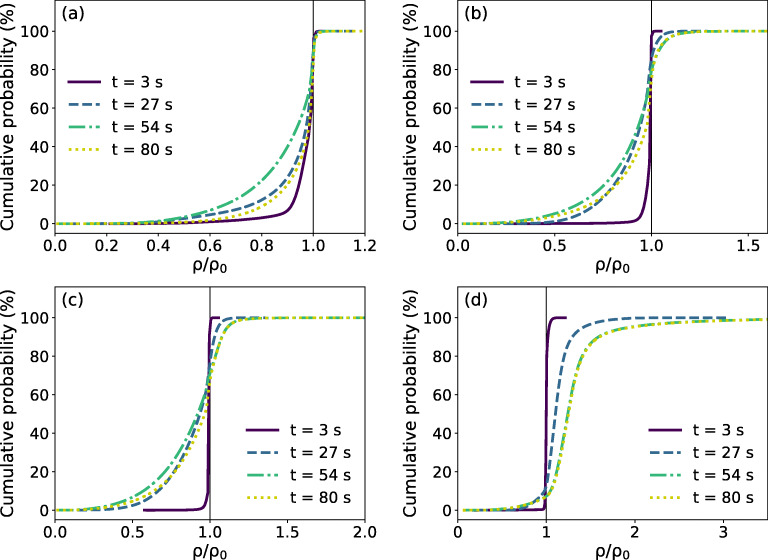
Cumulative probability of density ratio in **a** case I, **b** case II, **c** case III, and **d** case IV

Figure 6 demonstrates the cumulative probability of density ratio *ρ*/*ρ*_0_ at four typical time instants during the flow, where *ρ* and *ρ*_0_ are the current density and initial density of the snow, respectively. In MPM, each Eulerian particle (material point) denotes a piece of material with a certain volume, whose mass keeps constant. The density ratio of a particle can thus be derived from the deformation of the particle. By assuming small elastic deformation (Ortiz and Pandolfi 2004), the density ratio *ρ*/*ρ*_0_ can be estimated as ${e}^{{\left({\epsilon}_v^p\right)}_0-{\epsilon}_v^p}$ (see the derivation in the supplement).

The initial density ratio of snow is 1.0, illustrated by the black vertical lines in Fig. 6. A leftward shift of the curve reflects snow with increased volume, indicating that the snow is expanding under tension. On the contrary, a rightward shift represents snow with shrink volume, where the snow is under compression. At the beginning of the flow (t = 3 s), case I demonstrates a curve notably shifting leftward, reflecting the flow is spreading and in expansion. Similarly, cases II and III also show a leftward shifting curve, but the shifting is not as significant as that in case I. Indeed, the avalanches in cases II and III spread less, as a consequence of the higher friction and cohesion of the snow. In contrast to cases I∼III, the curve in case IV shifts more to the right side instead of to the left, which hints more snow in densification than in expansion. During the flowing process (from t = 3 s to t = 54 s), the avalanche in case I keeps being dominated by the spreading of the snow, as indicated by the curves shifting to the left in Fig. 6a. Both case II and case III illustrate snow in expansion (leftward shifting of the cumulative probability when *ρ*/*ρ*_0_ < 1) and in compaction (rightward shifting of the cumulative probability when *ρ*/*ρ*_0_ > 1). In contrast to case I, the avalanche in case IV shows the leading role of the compaction/densification of the snow, as implied by the curve shifting rightward in Fig. 6d. After the avalanches tend to stop (from t = 54 s to t = 80 s), the flows in cases I∼III are more densified, as the green dash-dot curves move rightward to the yellow dot ones in Fig. 6. It is noticed that the density ratio of the snow in case IV does not show visible variation when the avalanche tends to stop. This indicates that the densification of snow can saturate to a certain extent. As the avalanche has already been highly densified during the flowing process, no more densification occurs at the end. According to the density variation in Fig. 6, the snow granulation in cases II and III requires an appropriate combination of snow fracture and compaction. In comparison, the cohesionless flow in case I and the plug flow in case IV are primarily governed by expansion and compaction hardening, respectively.

**Fig. 7:**
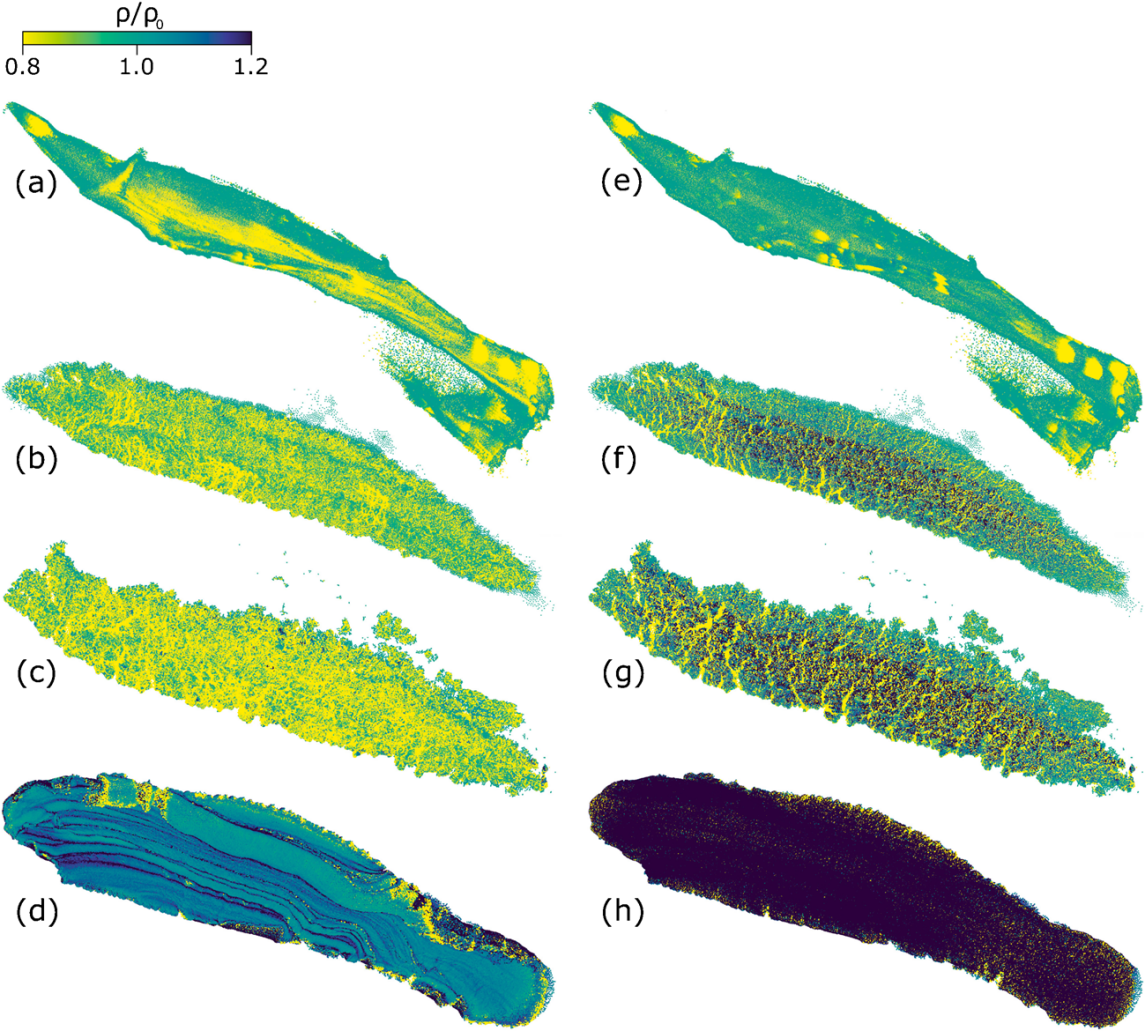
Typical distribution of density ratio in the four cases at t = 54 s. The left and right columns are the surface and bottom of the deposits, respectively. **a** and **e** case I; **b** and **f** case II; **c** and **g** case III; **d** and **h** case IV

To locate the snow particles in expansion and compaction, a typical spatial variation of density ratio in the four avalanches is illustrated in Fig. 7, where the left and right columns show the top and bottom views of the deposits, respectively. For all the four avalanches, the snow at the free surface generally has a smaller density ratio than that at the bottom. This variation along the flow depth direction is related to the increase of the pressure from the surface of the flow to its bottom, namely overburden pressure, which also plays a notable role in affecting the behavior of rock avalanches (Wang et al. 2015), landslides (Kawamura et al. 2007), and debris flows (Phillips 2006). As demonstrated in Fig. 7a and e, the snow in case I either remains its initial density or has a smaller one, consistent with the cumulative probability of density ratio in Fig. 6a. The distribution of the particles with different density ratios has no clear pattern neither along the streamwise direction nor in the transverse direction. The avalanches in case II (Fig. 7b and f) and case III (Fig. 7c and g) show similar patterns of the density ratio distribution. The snow particles at the avalanche surfaces mainly demonstrate densities smaller than their original ones. For the snow at the bottom layer, both density ratios smaller and larger than 1.0 are observed. Particularly, branches of snow particles with low density ratio (in yellow in Fig. 7f and g) form along the transverse direction of the flow, indicating discrete structures inside the flow. Compared to case III, case II has more snow particles with a density close to their initial density (in green) both at the free surface and the bottom of the avalanche. Unlike cases I∼III, case IV shows much more densified snow particles at the free surface and the bottom layer of the avalanche. Interestingly, compacting shear lines formed by highly densified snow (in Fig. 7d) are captured along the streamwise direction at the free surface.

#### Avalanche deposition

**Fig. 8:**
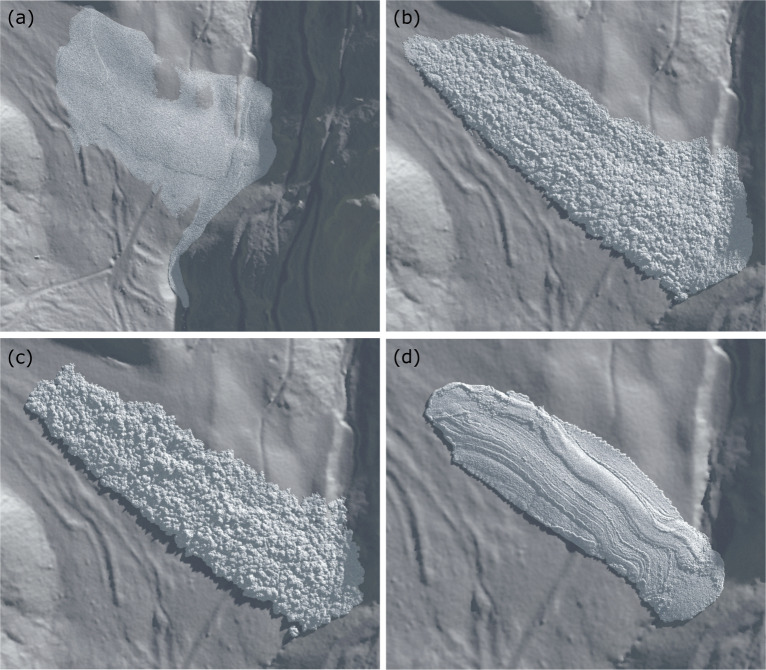
Final deposits of the four flows on the real terrain. **a** case I; **b** case II; **c** case III; **d** case IV

The final deposits of the four avalanches are shown in Fig. 8. The average deposit heights from case I to case IV are 1.3 m, 2.3 m, 3.1 m, and 2.8 m, respectively. The avalanche in case I stops as a dry cohesionless granular flow, where the surface of the deposit is smooth compared to the other three cases. Moreover, the surface area of the deposited mass is relatively large due to notable spreading of the cohesionless snow particles. This deposition feature is consistent with the initial flow characteristic observed in Fig. 4a, where the snow sample has evolved into a cohesionless granular flow. In contrast, both the avalanches in cases II and III have granules formed at the surface of the deposits (Fig. 8b and c), and the slabs developed at the initial stage of the flows (Fig. 4b and c) disappear. Nevertheless, the size of the deposit granules and the roughness of the deposit surface appear to be related to the fractures developed at the initial stage of the avalanche. The less the initial fractures and the bigger the broken pieces, the larger the deposit granules and the rougher the deposit surface. Compared with case II, the more cohesion and higher tensile strength in case III lead to the less fractures and larger broken pieces in Fig. 4c as well as the larger granules and rougher deposit surface in Fig. 8c. The final deposit in case IV shows a surface composed of multiple strips along the streamwise direction as illustrated in Fig. 8d. The compacting shear lines between the strips are formed by highly densified particles as indicated in Fig. 7d. The surface roughness of all the avalanches can be found in the supplement.

### Experimental comparison

The snow avalanche happened on 7 February 2003 at Vallée de la Sionne is used as a benchmark case. There are mainly two reasons for the selection of this particular avalanche. First, the entrainment depth was small. The avalanche was artificially triggered 2 days after a naturally released avalanche. As the natural avalanche entrained the majority of the snow cover, the entrainment by the artificially triggered avalanche was very limited, which offers the basis to be compared to our simulation in which entrainment is not considered. Second, the data of the avalanche were well documented from its release to its deposition, including front velocity and flow path. The density profile of an avalanche deposit was measured from an avalanche that was released shortly before the avalanche on 7 February 2003. Both avalanches were speculated to have extremely similar snow conditions. According to field observation, the avalanche on 7 February was a cold avalanche. Thus, relatively low snow friction and cohesion (*M* = 0.7; *β* = 0.2) are adopted in the simulation. The initial snow density of 200 kg/m^3^ refers to the field data in (Sovilla et al. 2006). Detail parameters are summarized in Table 1. The video of case V can be found in the supplement.

**Fig. 9:**
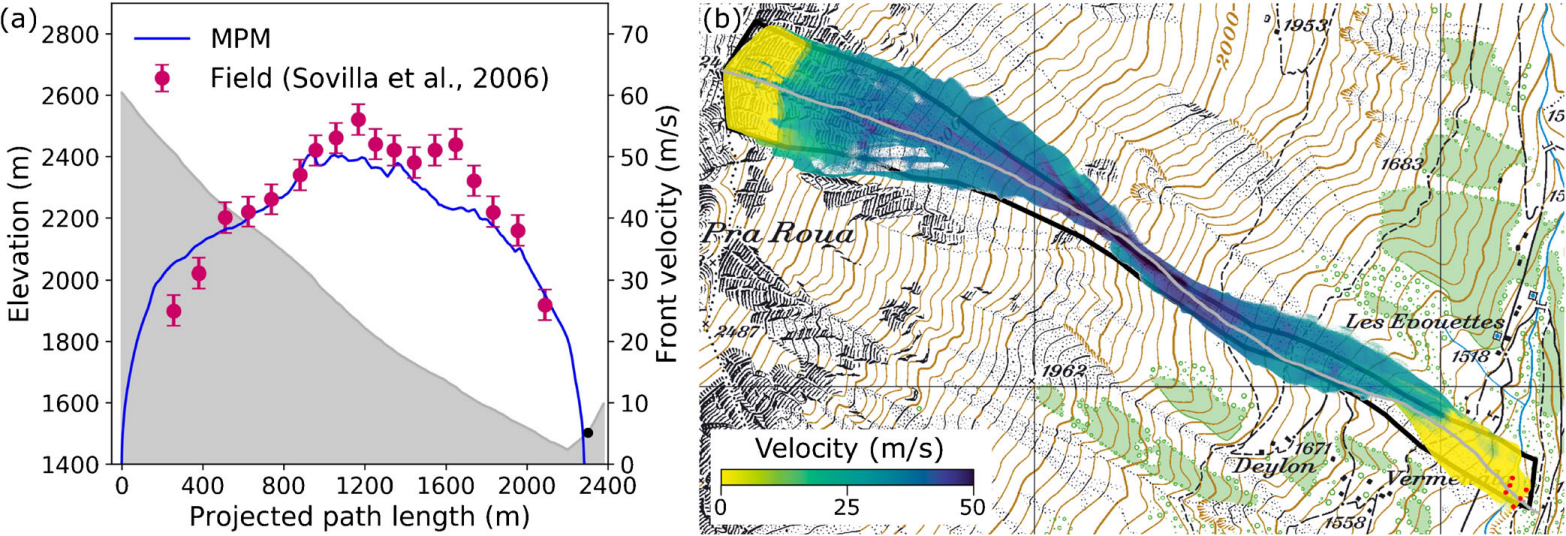
**a** Front velocity evolution along the flow path denoted by the gray curve, on which the black dot shows the observed stopping point of the avalanche; **b** flow path of the simulated snow avalanche colored by flow velocity, in comparison with the real one in solid line. The gray curve demonstrates the avalanche path in **a**

Figure 9 shows the front evolution and flow path from the field measurements and the MPM simulation case V. The evolution of front velocity along the flow path is illustrated in Fig. 9a. The front velocity from the field was obtained by video analysis (Sovilla et al. 2006). The MPM result is collected by excluding 1% of the particles at the front of the avalanche to diminish the effect of scattered particles from the main body (Li et al. 2020). As illustrated in Fig. 9a, the agreement between the MPM and the field data is largely satisfactory. It is noticed that the MPM model overestimates the front velocity at the beginning of the flowing process and underestimates the maximum front velocity, which might be related to the neglection of entrainment. The slight discrepancy of the velocity can also be speculated from the marginal difference of the flow path in Fig. 9b. The velocity obtained in the MPM simulation significantly depends on the bed friction coefficient, which is back calculated as 0.49 in case V to get the reasonable consistency.

The flow path of the simulated avalanche in case V is compared to that recorded from the field in Fig. 9b. The flow path from MPM is obtained by superposing flow profiles with an interval of 3.3 s (10 frames in the simulation) and is colored by flow velocity. The field measurement is denoted in solid line in Fig. 9b. In the MPM simulation, the smallest flow width occurs at the narrowest flow channel bounded by lateral bumps, which contributes to the appearance of the large flow velocity. Compared to the field data, the MPM result generally gives a wider flow, which might be due to the cold nature of the simulated avalanche and the extensive lateral spreading of snow particles. It should be noted that the flow path from the field in Fig. 9b only accounts for the dense part of the cold snow avalanche, while the dilute powder cloud was neglected. This may exclude snow particles at the edge of the dense part and lead to the smaller flow width compared to that of the simulated avalanche.

**Fig. 10:**
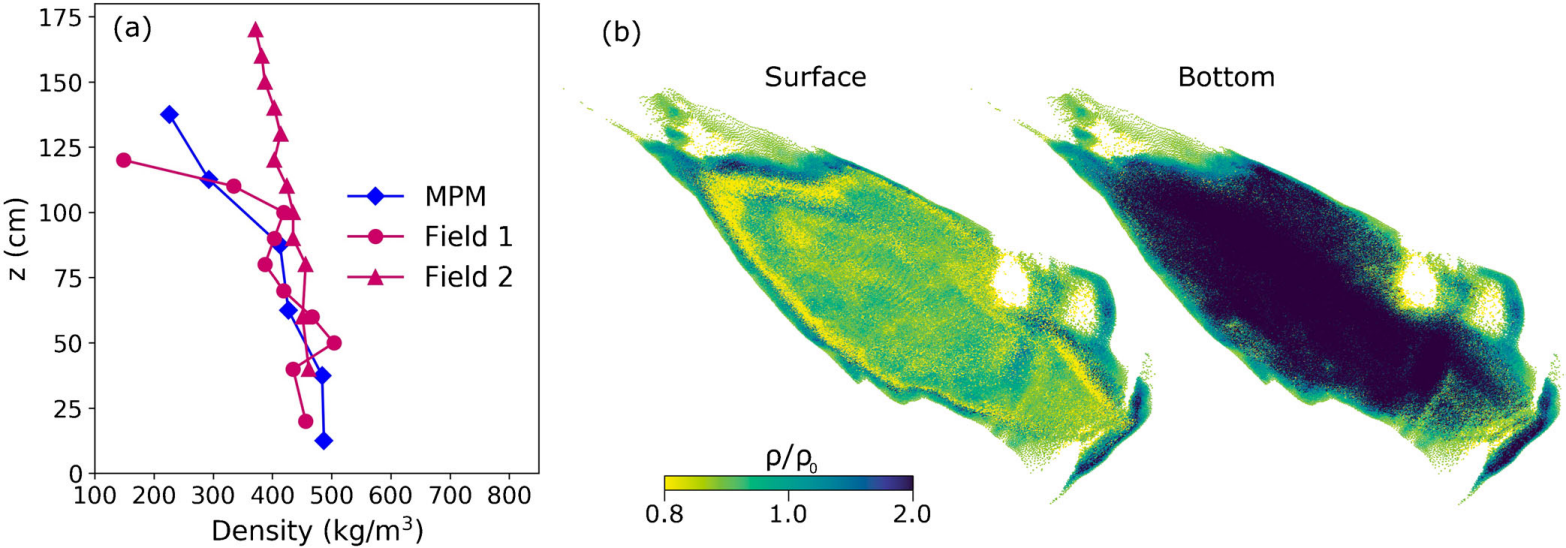
**a** Density profile at the tip of the avalanche deposit, *z* is along the direction of negative gravity, and *z* = 0 corresponds to the bottom of the measured point; **b** density distribution at the surface and bottom of the avalanche deposit in case V

In addition to the front velocity and flow path, the density profile of the avalanche deposit from MPM is compared with the field measurement as shown in Fig. 10a. The two field measurements were taken at two different locations in the avalanche deposit. Both locations were at the tip of the avalanche deposit since it was dangerous to enter the slope. The specific locations of the measurements were not recorded. Accordingly, the MPM result in Fig. 10a is extracted at the tip of the simulated avalanche. In particular, the density profiles at six points at the tip of the avalanche deposit (in red in Fig. 9b) are used to get the averaged density profile in Fig. 10a. It should be noted that the results in Fig. 10a is not for quantitative comparison, since the locations of the field measurement points are very likely different from the six points in the MPM simulation. Nevertheless, we get a generally consistent density profile. The density variation is closely related to the hardening factor *ξ*. The smaller the *ξ*, the more the snow compaction, and the higher the snow density of the avalanche deposit. This can also be conjectured from cases I to IV, as the most significant snow compaction happens in case IV where *ξ* is the smallest. The density distribution at the surface and bottom of the avalanche deposit is illustrated in Fig. 10b. Similar to the deposits of cases I∼IV in Fig. 7, the density at the surface of the deposit is smaller than that at the bottom, agreeing with the distribution of the overburden pressure.

## Discussion

With reference to the classification of real snow avalanches based on their release, the avalanches in cases II and III can be regarded as slab avalanches. It should be noted that the simulations in this study do not model the entire snow cover on the terrain for computational efficiency. Thus, the release mark, a fracture line or a point release, cannot be explicitly observed. Nevertheless, the flow features shortly after the release (as shown in Fig. 4) provide clear indications on the release type. The avalanche in case I collapses as a dry cohesionless granular flow, which is more consistent with the point release in comparison to the fracture line. In reality, the plug flow simulated in case IV generally does not start from the release zone and normally forms at a later stage of an avalanche when it reaches the lower and warmer part of the mountain (Köhler et al. 2018a). It is worth noting that this study aims at examining the distinct flow features with a wide variety of snow types/properties, while other conditions (i.e., release size, release position, terrain) are fixed to be exactly identical. By changing snow properties during the flowing process of an avalanche, the MPM tool can be used to obtain expected flow regime transitions in real snow avalanches (e.g., from cold dense to warm plug), but is not the focus here.

The four flow regimes identified for dense snow avalanches from the field include cold dense, warm shear, sliding slab, and warm plug (Köhler et al. 2018b). The simulated avalanche in case I can be characterized as a cold dense regime, since it behaves as a cohesionless granular flow without the occurrence of granules or snow clods. This identification agrees with the 2D simulation (Li et al. 2020). The snow properties in case II and case III gave notably different regimes in the 2D modeling, while they lead to similar flow regimes in the 3D cases. As illustrated in the “Results” section, both the avalanches in cases II and III are initially in the sliding slab regime reflected by the multiple blocks sliding down the terrain and then transform to the warm shear regime as granules occur. The difference of the flow behaviors in the 2D and 3D simulations is closely related to the distinct terrains adopted. An ideal slope was applied in the 2D study, while a complex terrain is used in this 3D investigation. The observed difference hints the effect of terrain irregularity on the flow regime transition. Indeed, a more complex terrain can induce significant local velocity variations, leading to a different rheology (Gaume et al. 2020). The avalanche simulated in case IV is apparently a plug flow as identified in the 2D modeling, which shares similarity with real avalanches in the warm plug regime.

The feature of snow avalanche deposits can offer indications on their flow regimes. (Issler et al. 2020) identified three types of avalanche deposits (i.e., blocky and sharply bounded deposit, snow clods embedded in fine-grained snow, and fine-grained snow with small/no snow clods) and associated them with three flow regimes (i.e., dense flow, fluidized flow, and suspension flow), respectively. From the simulations of dense snow avalanches in this study, distinct textures of avalanche deposits have been obtained without consideration of the fluidized and suspension flow regions. This observation entails careful distinction of different deposit textures of avalanches even in the same flow regime (e.g., dense flow in this study). Note that the motions of fluidized flow and suspension flow are dominated by different physical processes (e.g., particle collision and friction, particle-air interaction) from the dense flow investigated in this study. Further consideration of the fluidized and suspension flows will require implementation of additional constitutive laws and interactions between the different flows.

Snow granules/clods are noticed at the surface of the avalanche deposits in this study. Four sources of snow granules were proposed in (Issler et al. 2020), including (1) external source like snow on trees, (2) accretion in totally inelastic collisions (Steinkogler et al. 2015), (3) remnants of the unbroken released slab, and (4) pieces of the snow cover. In this study, the snow granules appear to result from broken slab pieces, rounding through inelastic collisions as there are no external source and no snow cover. Similar deposit surfaces have been noticed from other types of gravitational mass movements. For example, the deposit surfaces of rock avalanches also have large particles (e.g., cobbles and gravels) because of segregation (Ren et al. 2018).

A complex real terrain at Vallée de la Sionne is applied in this study, which recovers the natural boundary condition of the simulated snow avalanches. Based on the simulation data, it is found that the terrain feature may affect local flow behaviors especially at the early stage of the avalanche. For example, the developed branches in case I in Fig. 4a follow the concave parts of the terrain. Nevertheless, the local flow characteristics during the flow show weak/no correlation to local terrain features. For instance, the density distribution within the flows in Fig. 7 has no direct relation to the local curvature and slope angle of the terrain. Throughout the entire flowing process of the avalanches, both the snow properties and the terrain geometry may control and change the avalanche behavior. At the early stage, it is easier to get correlation between local flow features and local terrain textures. As the avalanches flow down the terrain, the effect of the passed terrain will accumulate, which makes the correlation weaker.

The key novelty of this study is a sophisticated 3D elastoplastic model that can simulate, over complex topography, different regimes of snow avalanches with unprecedented details. Compared with depth-averaged approaches predominantly adopted in existing studies of real-scale avalanches, the true 3D modeling in this study enables us to capture crucial processes including snow fracture, snow granulation, and snow densification in all directions of interest. Concerning constitutive modeling, existing numerical studies of snow avalanches usually assume a Voellmy fluid model or viscoplastic laws (e.g., Bingham model, Herschel-Bulkley model) that are developed based on macro flow behavior of snow avalanches (Eglit et al. 2020; Kern et al. 2004). For such rheological models, rigid behavior is commonly assumed when the stress is below a pure shear-based yield criterion. In contrast, our model accounts for snow elasticity and mixed-mode failure. Here, it is the collective behavior of individual snow particles with varying properties that naturally gives rise to distinct macroscopic flow characteristics of snow avalanches including the viscous behavior. Similar strategies were adopted in modeling other gravitational flows such as mud flows and landslides. For example, Zhang et al. (2019) studied a landslide with PFEM and an elastoviscoplastic model with strain softening. (Prime et al. 2014) investigated a mud flow by adopting an elastoplastic relation and a viscous Bingham law before and after soil failure, respectively.

It should be noted that the 3D modeling in this study inevitably increases the computational cost compared to 2D modeling and may be extremely expensive when a large amount of snow avalanches need to be simulated and analyzed. In addition, the adopted constitutive model does not consider variations of snow properties with time and temperature (e.g., sintering (Szabo and Schneebeli 2007)). Moreover, this study focuses on dense snow avalanches without consideration of the cloud layer of snow avalanches. With a new constitutive law for the cloud, a powder snow avalanche could be studied with MPM by considering the cloud-dense core interaction and the cloud-air interaction. Despite the limitations, the MPM framework has been proven as a promising and physically based numerical tool for investigating snow avalanches. Although not considered in this study, entrainment in snow avalanches can indeed be modeled with MPM and will be explored in the future, by either explicitly putting snow particles on the terrain or adjusting mass and momentum conservation according to empirical/theoretical prediction of entrained mass. Spatial variability of properties (e.g., friction) of the released snow (Schweizer et al. 2008) and the slope (Yang et al. 2019) could also be further considered with reference to field data in future study. In addition to snow avalanches, MPM has great potential to deal with other gravitational mass movements, including rock avalanches and debris flows. As the constitutive model in this study is developed for snow, additional constitutive models may need to be implemented to capture the behavior of rock and soil, such as Mohr-Coulomb model (Ceccato et al. 2018; Soga et al. 2016), Drucker-Prager model, and *μ*(*I*) rheological relation (Gaume et al. 2011; Xu et al. 2019). Furthermore, more field data need to be collected in the future for extensive validation of the 3D simulations.

## Conclusions

Using the 3D material point method and the elastoplastic constitutive law, this study explores distinct behaviors and different regimes of real-scale snow avalanches with unprecedented details. The implementation of the terrain of Vallée de la Sionne recovers the natural boundary conditions of the avalanches. The 3D modeling fully captures snow avalanche features in all directions of interest. Key characteristics of the avalanches from release to deposition, including the failure pattern after release, the density variation during the flow, and the deposit surface, have been analyzed for deeper understanding and better identification of avalanches in different regimes.

Four snow avalanches have been modeled to characterize the different regimes, including a cohesionless granular avalanche, two avalanches initially behaving as sliding slabs then demonstrating features of warm shear flows, and an avalanche moving as a plug flow. Both temporal and spatial variations of the dynamic behavior of the avalanches have been explored, showing distinctive features of the different flow regimes. In particular, brittle and ductile fractures have been identified after the release of the avalanches, which correspond to various amounts of plastic particles in the avalanches and contrasting stress state of the plastic particles. In comparison to the brittle fracture where snow particles fail under compression and shear and under tension and shear, the ductile fracture is primarily contributed from particles failed in tension and shear. In addition, the density variation of the avalanches reveals their extension and densification as a result of volume expansion and shrinkage, respectively. Due to the snow weight, the density appears naturally larger at the bottom of the avalanches compared to that at the surface. Interesting structures along both the transverse and streamwise directions of the avalanches have been identified from the avalanche density distribution, highlighting the importance of 3D consideration for detailed and accurate analyses of snow avalanches. Furthermore, the deposits of the four avalanches have been examined, where their surfaces with distinct characteristics have been attained, including a smooth surface, two surfaces with granules, and a surface with compacting shear planes.

Moreover, we have back calculated an avalanche happened at Vallée de la Sionne and benchmarked the MPM modeling. Reasonable agreement is observed between the simulation results and the data from the field. It has been demonstrated that the MPM framework is capable of capturing key features of different 3D real-scale snow avalanches on a complex terrain, which serves as a promising tool for analyzing both temporal and spatial variations in dynamic behavior of avalanches. With further implementation of modified constitutive laws, the presented computational framework will be able to tackle other types of gravitational mass movements.

## Supplementary information

Video 1 (MP4 7802 kb)

Video 2 (MP4 4897 kb)

Video 3 (MP4 4508 kb)

Video 4 (MP4 3540 kb)

Video 5 (MP4 4398 kb)

ESM 6 (PDF 84.2 kb)

ESM 7 (PDF 93.7 kb)
