# Supplementary material for: Three-dimensional and real-scale modeling of flow regimes in dense snow avalanches
Source: Landslides. 2021 Jul 29;18(10):3393–406. doi: 10.1007/s10346-021-01692-8 (PMC8550512; doi:10.1007/s10346-021-01692-8)
Supplement: Supplementary file 6 — (PDF 84.2 kb) [file 10346_2021_1692_MOESM6_ESM.pdf]

Noname manuscript No.
(will be inserted by the editor)

Supplementary material for: Three dimensional and real-scale modeling of flow regimes in dense snow avalanches

Xingyue Li · Betty Sovilla · Chenfanfu Jiang · Johan Gaume

Received: date / Accepted: date

1 Effect of grid size and grid orientation

Using the model setup of case V detailed in the paper, three cases with different grid sizes ($dx = 0.5$ m, 0.35 m, 0.25 m) were simulated. The front evolution, density distribution and flow path with the different dx are demonstrated in Figs. 1∼3 below. The front velocity from the MPM simulations in Fig. 1 is calculated by excluding 1% of the particles at the front of the avalanches to diminish the effect of scattered particles from the main body. The density distribution in Fig. 2 is extracted from the tip of the avalanche deposit. The field results were from two different locations. The MPM results are the averaged density distribution from six points. “MPM” in Figs. 2a&2b and “MPM 1” in Fig. 2c are obtained from the same six points. “MPM 2” in Fig. 2c is attained from different points. The flow path from MPM in Fig. 3 is obtained by superposing flow profiles with an interval of 3.3 s (10 frames in the simulation), and is colored by flow velocity.

As shown in Figs. 1&3, the front velocity and flow path do not vary much when dx is changed. In comparison, the density distribution in Fig. 2 does show

X. Li
School of Architecture, Civil and Environmental Engineering, Swiss Federal Institute of Technology, Lausanne, Switzerland

B. Sovilla
WSL Institute for Snow and Avalanche Research, SLF, Davos, Switzerland

C. Jiang
Computer and Information Science Department, University of Pennsylvania, Philadelphia, USA

J. Gaume (Corresponding author)
School of Architecture, Civil and Environmental Engineering, Swiss Federal Institute of Technology, Lausanne, Switzerland
WSL Institute for Snow and Avalanche Research, SLF, Davos, Switzerland
Tel.: +41-21 693 51 69
E-mail: johan.gaume@epfl.ch

differences. It is noticed that, for the same six points, the density distribution could be different with the different dx. But the trends are similar (density increases with snow depth). The density distribution from the simulation is not for quantitative comparison, since the locations of the six points are very likely different from the locations of the field measurement. By adjusting the locations for outputting the numerical result, more consistent result can be obtained when $dx = 0.25$ m. For example, “MPM 2” in Fig. 2c is the averaged result of different points compared with “MPM 1” in Fig. 2c, and “MPM 2” has a better agreement with the field data.

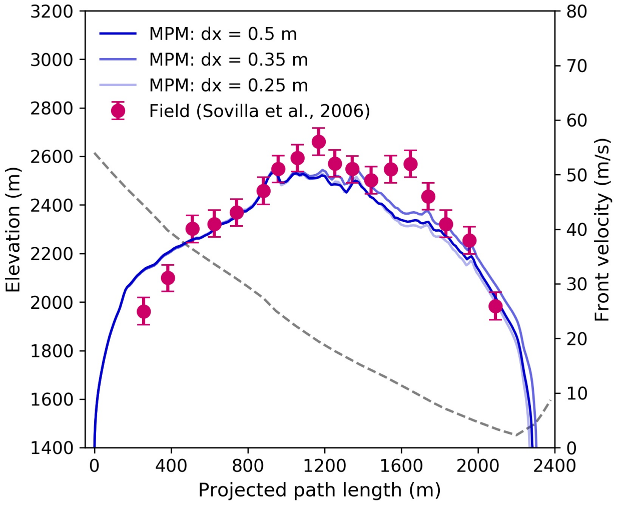

Fig. 1 Front velocity along the flow path in gray with different dx.

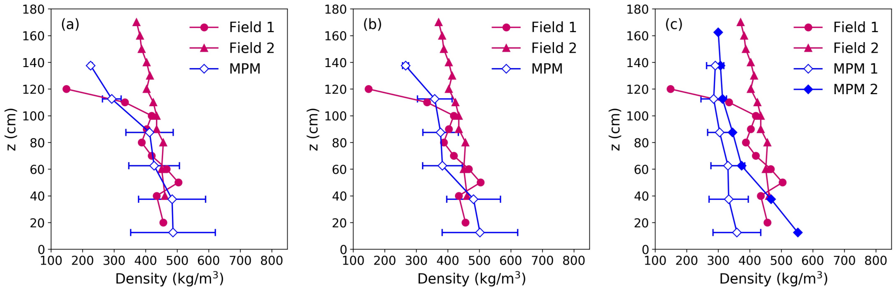

Fig. 2 Density distribution with (a) $dx = 0.5$ m, (b) $dx = 0.35$ m, (c) $dx = 0.25$ m.

It should be noted that the effect of grid size on front velocity and flow path is trivial, because the front velocity and flow path are relatively macroscopic characteristics. In comparison, the density distribution is a local quantity,

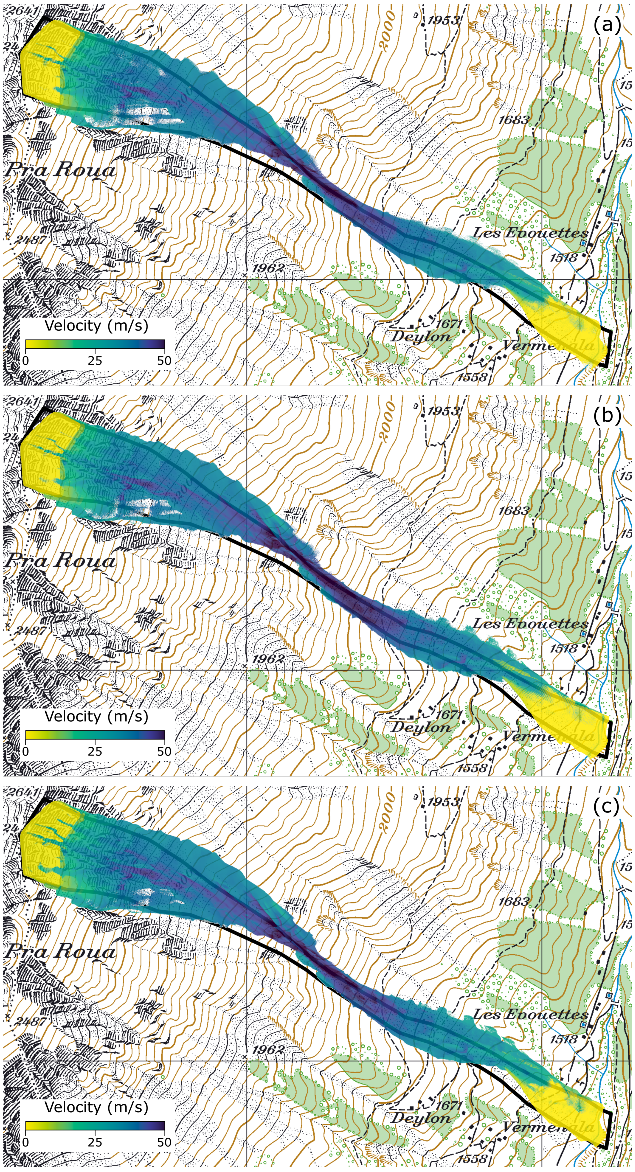

Fig. 3 Flow path with (a) $dx = 0.5$ m, (b) $dx = 0.35$ m, (c) $dx = 0.25$ m.

which is more sensitive to the change of the setup and is expected to have more variations.

To study the effect of grid orientation, we rotate the setup (i.e. terrain and released snow) instead of rotating the background grid, as demonstrated in Fig. 4 below. Fig. 4 shows the front view of the setups, where the terrain and the released zone are in gray and red, respectively. Fig. 4a shows the default

grid orientation used in the manuscript. In Figs. 4b&4c, the setup is rotated 22.5° and 45° (i.e. $\theta = 22.5°$ and $\theta = 45°$), respectively.

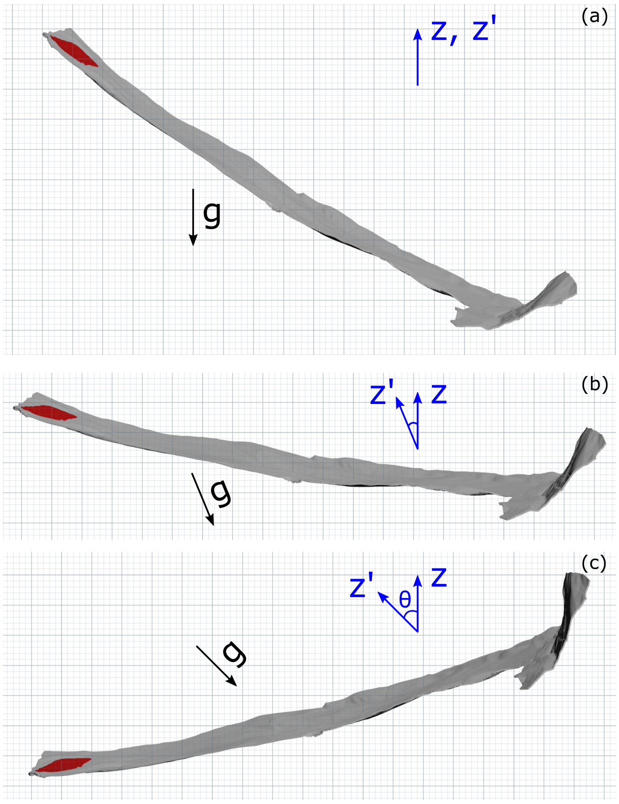

Fig. 4 Setup of three simulations with different mesh orientations. g is the direction of gravity. z and z' denote the original and rotated axes along the negative gravity direction. θ is the rotation angle. (a) $\theta = 0°$, (b) $\theta = 22.5°$, (c) $\theta = 45°$.

The front velocity, density distribution and flow path from the three cases in Fig. 4 are illustrated in Figs. 5∼7 below. From Figs. 5&7, the front velocity and flow path of the three cases with different mesh orientations are almost identical. More notable discrepancy is observed from the density distribution in Fig. 6. As discussed above, the density distribution contains more local details, and is expected to have more variations with the change of the setup. Generally speaking, the effect of mesh orientation on the front velocity, density distribution, and flow path is not significant.

From the above investigation, we conclude that grid size and grid orientation do not significantly affect the simulation results.

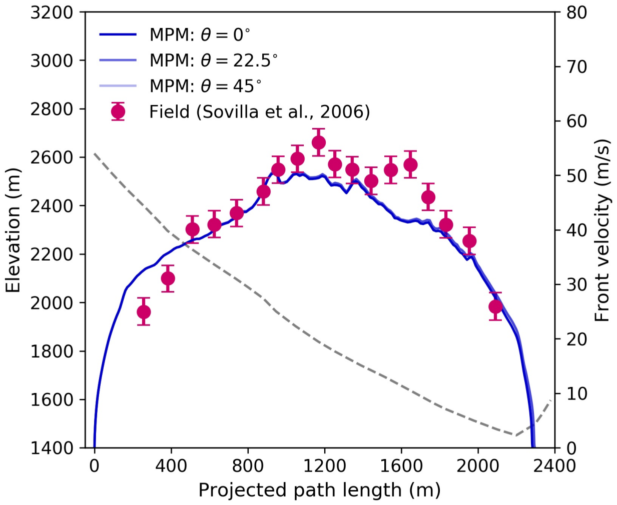

Fig. 5 Front velocity along the flow path in gray with different grid orientations.

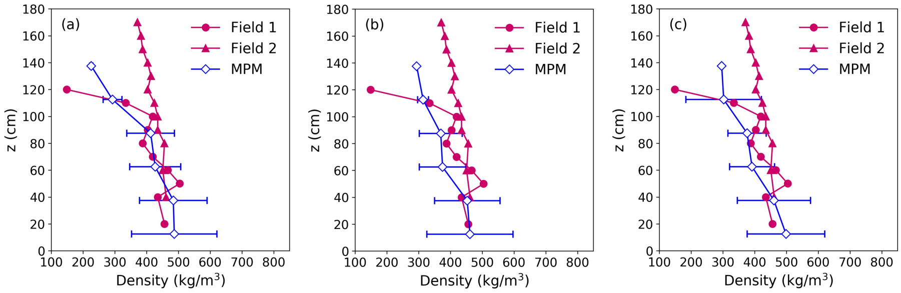

Fig. 6 Density distribution with (a) $\theta = 0°$, (b) $\theta = 22.5°$, (c) $\theta = 45°$.

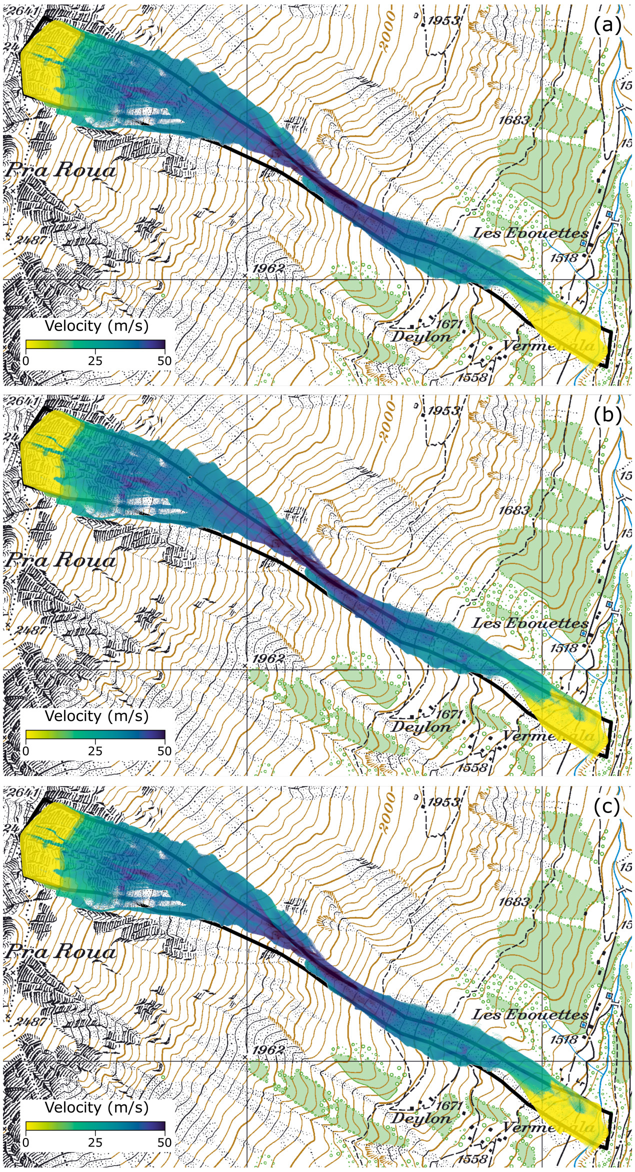

Fig. 7 Flow path with (a) $\theta = 0°$, (b) $\theta = 22.5°$, (c) $\theta = 45°$.

2 Derivation of density ratio

According to mass conservation,

$$m = m_0 \tag{1}$$

where m_0 and m are the initial mass and current mass of snow, respectively. Denoting the mass by density and volume,

$$\rho V = \rho_0 V_0 \tag{2}$$

where V_0, ρ_0 and V, ρ are the volume and density of snow at the initial and current time instants, respectively. From Eq. 2,

$$\frac{\rho_0}{\rho} = \frac{V}{V_0} \tag{3}$$

With the assumption that elastic deformation is small [1],

$$\hat{J}^p = \frac{V}{V_0} \tag{4}$$

where $\hat{J}^p$ quantifies the plastic volume change such that $\hat{J}^p = 1$ denotes no volume change, $\hat{J}^p < 1$ and $\hat{J}^p > 1$ represent volume decrease and volume increase, respectively. Substituting $\hat{J}^p = J^p/J_0^p$ into Eq. 4,

$$\frac{J^p}{J_0^p} = \frac{V}{V_0} \tag{5}$$

where $\log(J^p) = \epsilon_v^p$ and ϵ_v^p is the volumetric plastic strain [2]. In addition, $\log(J_0^p) = (\epsilon_v^p)_0$ and $(\epsilon_v^p)_0$ is the initial volumetric plastic strain. From Eq. 5,

$$\frac{e^{\epsilon_v^p}}{e^{(\epsilon_v^p)_0}} = \frac{V}{V_0} \tag{6}$$

Substituting Eq. 3 into Eq. 6,

$$\frac{e^{\epsilon_v^p}}{e^{(\epsilon_v^p)_0}} = \frac{\rho_0}{\rho} \tag{7}$$

The density ratio can then be expressed as

$$\frac{\rho}{\rho_0} = e^{(\epsilon_v^p)_0 - \epsilon_v^p} \tag{8}$$

It should be noted that the volumetric plastic strain ϵ_v^p of a particle (a material point) in MPM is calculated based on its surrounding particles. The reduction in ρ/ρ_0 indicates volume expansion, spreading, and fracture, while the increase in ρ/ρ_0 hints densification and compaction.

3 Supplementary figures of terrain and avalanche deposits

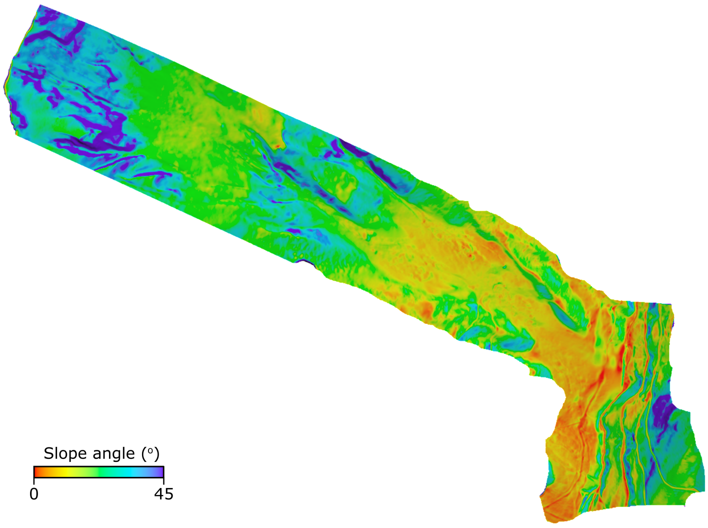

Fig. 8 Slope angle map of the modeled terrain.

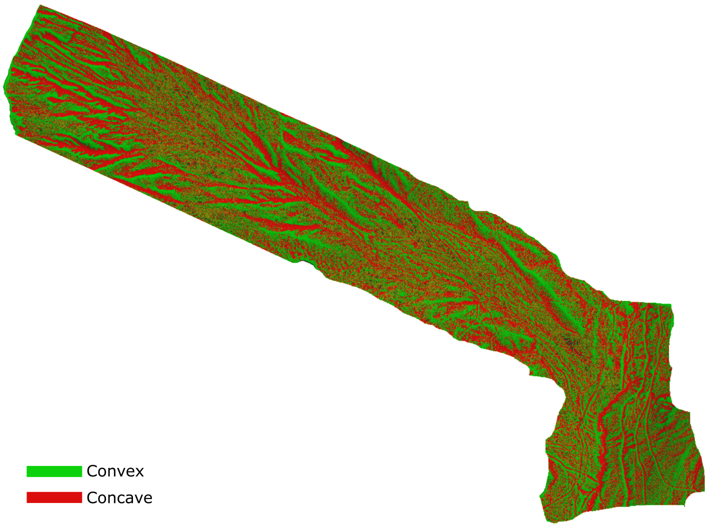

Fig. 9 Curvature map of the modeled terrain.

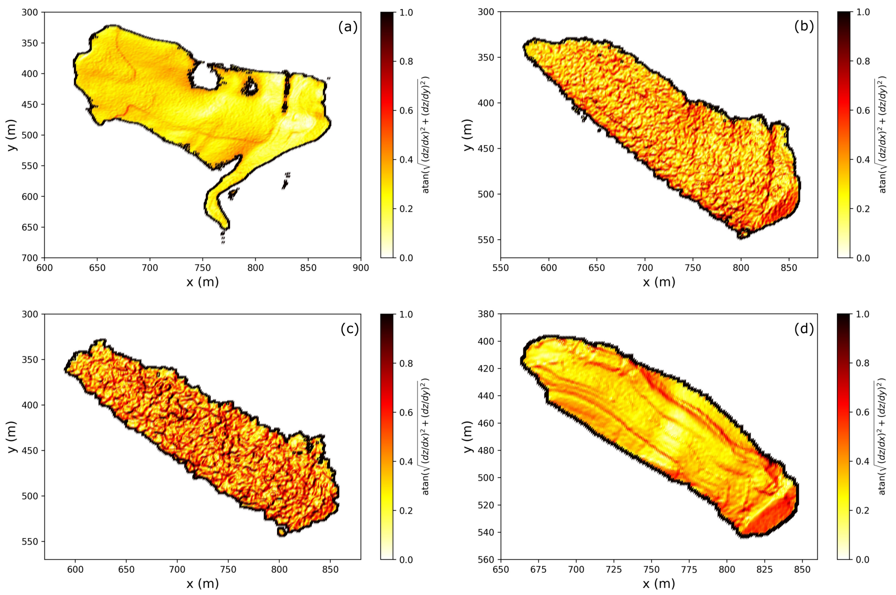

Fig. 10 Surface roughness of avalanche deposits in (a) Case I; (b) Case II; (c) Case III; (d) Case IV. The x, y, z in the calculation of the surface roughness are the coordinates of snow particles at the surfaces of the avalanche deposits.

References

1. Ortiz M, Pandolfi A, A variational Cam-clay theory of plasticity, *Computer Methods in Applied Mechanics and Engineering*, 193(27-29), 2645–2666 (2004)
2. Gaume J, Gast T, Teran J, van Herwijnen A, Jiang C, Dynamic anticrack propagation in snow, *Nature Communications*, 9(1), 1–10 (2018)
