# Supplementary material for: Three-dimensional and real-scale modeling of flow regimes in dense snow avalanches
Source: Landslides. 2021 Jul 29;18(10):3393–406. doi: 10.1007/s10346-021-01692-8 (PMC8550512; doi:10.1007/s10346-021-01692-8)
Supplement: Supplementary file 7 — (PDF 93.7 kb) [file 10346_2021_1692_MOESM7_ESM.pdf]

Supplementary videos for “Three dimensional and real-scale modeling of flow regimes in dense snow avalanches”

Xingyue Li[1], Betty Sovilla[2], Chenfanfu Jiang[3], and Johan Gaume[1,2]

[1]School of Architecture, Civil and Environmental Engineering, Swiss Federal Institute of Technology, Lausanne, Switzerland
[2]WSL Institute for Snow and Avalanche Research, SLF, Davos, Switzerland
[3]Computer and Information Science Department, University of Pennsylvania, Philadelphia, USA

Correspondence: Johan Gaume (johan.gaume@epfl.ch)

Video 1. The snow avalanche in Case I colored by velocity (left panel) and colored white (right panel). The legend of the velocity is in Fig. 9(b) of the manuscript.

Video 2. The snow avalanche in Case II colored by velocity (left panel) and colored white (right panel). The legend of the velocity is in Fig. 9(b) of the manuscript.

Video 3. The snow avalanche in Case III colored by velocity (left panel) and colored white (right panel). The legend of the velocity is in Fig. 9(b) of the manuscript.

Video 4. The snow avalanche in Case IV colored by velocity (left panel) and colored white (right panel). The legend of the velocity is in Fig. 9(b) of the manuscript.

Video 5. The snow avalanche in Case V colored by velocity (left panel) and colored white (right panel). The legend of the velocity is in Fig. 9(b) of the manuscript.
